# Supplementary material for: Antibiotic-Induced Cell Chaining Triggers Pneumococcal Competence by Reshaping Quorum Sensing to Autocrine-Like Signaling
Source: Cell Rep. 2018 Nov 27;25(9):2390–2400.e3. doi: 10.1016/j.celrep.2018.11.007 (PMC6289044; doi:10.1016/j.celrep.2018.11.007)
Supplement: Document S1. Figures S1–S7 and Tables S1–S6 [file mmc1.pdf]

Cell Reports, Volume *25*

Supplemental Information

Antibiotic-Induced Cell Chaining Triggers Pneumococcal Competence by Reshaping Quorum Sensing to Autocrine-Like Signaling

Arnau Domenech, Jelle Slager, and Jan-Willem Veening

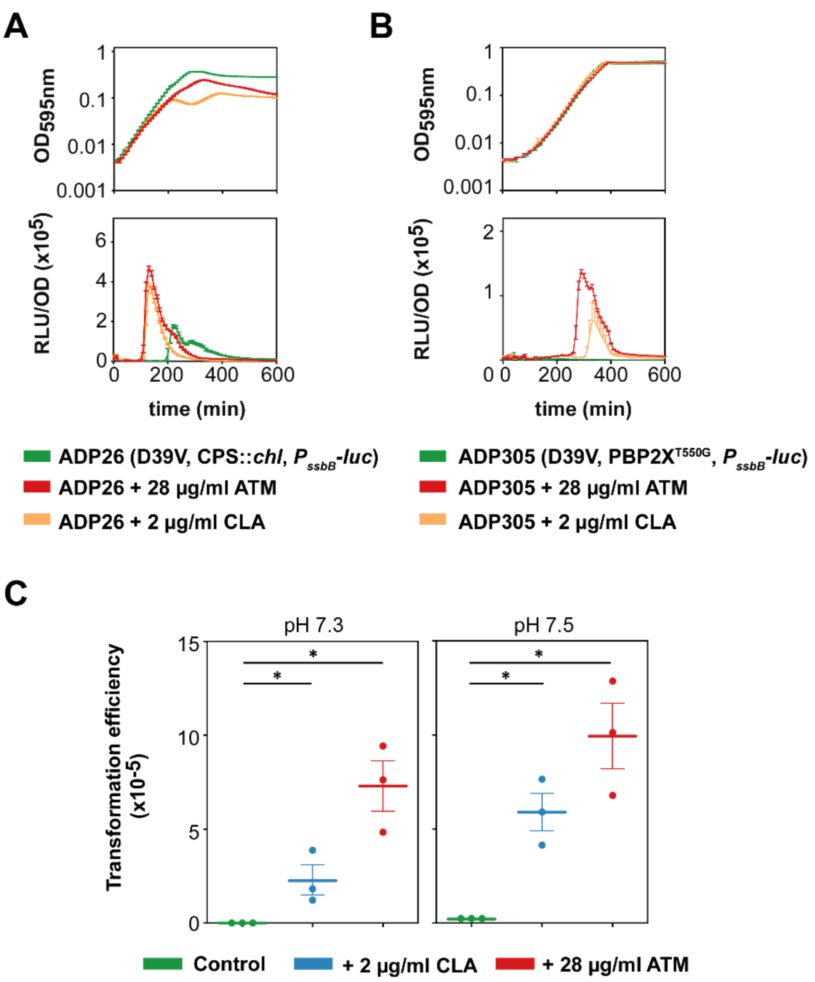

Figure S1. A) Competence induction in an unencapsulated variant of D39V. Both aztreonam (ATM) and clavulanic acid (CLA) are able to promote competence, as observed for the encapsulated strain, when grown in C+Y (pH 7.3). Note that unencapsulated strains can induce natural competence at lower pH than the parental encapsulated strain (Moreno-Gámez et al., 2017), explaining bioluminescence activity at pH 7.3, which is non-permissive for encapsulated strains in the absence of antibiotics. Average of 3 replicates and Standard Error of the Mean (SEM) are plotted. **B) Competence induction in the ADP305 mutant with reduced susceptibility to betalactams ($PBP2X^{T550G}$).** Cells were grown in C+Y at competence-permissive pH 7.3 The average of 3 replicates and Standard Error of the Mean (SEM) are plotted for each condition. Despite the reduced susceptibility to betalactams, both ATM and CLA were able to induce competence, as observed for the wild-type. Average of 3 replicates and Standard Error of the Mean (SEM) are plotted. **C) Induction of horizontal gene transfer by ATM and CLA.** DLA3 (tetracycline resistant) and MK134 (kanamycin resistant), were individually grown to OD_{595nm} 0.4 in C+Y pH 6.8 at 37°C. Then, a mixed 100-fold dilution of both strains were grown in C+Y pH 7.3 (non-permissive conditions) or pH 7.5 (permissive conditions) to OD_{595nm} 0.3 to promote the transfer of genes. Antibiotics were added where indicated (2 µg/ml clavulanic acid or 28 µg/ml aztreonam). Afterwards, serial dilutions of cultures were plated with or without antibiotics (for the recovery of the total viable counts) and with the combination of 250 µg/ml of kanamycin plus 1 µg/ml tetracycline, and the ratio between total viable cells and transformants was calculated. *Statistically significant more transformants than wild-type, mean comparison test $p<0.05$. Three replicates, average and Standard Error of the Mean (SEM) are plotted. Related to Figure 1.

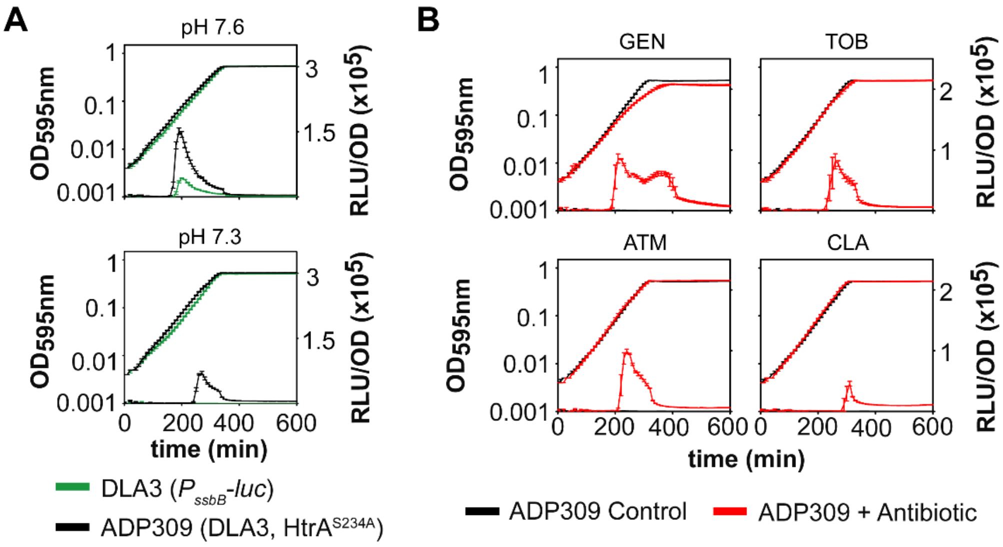

Figure S2. A) Growth curves (OD_{595}) and bioluminescence activity (RLU/OD_{595}) of wild-type and $HtrA^{S234A}$ strains. Strains DLA3 (P_{ssbB}-*luc*; green lines) and ADP309 (P_{ssbB}-*luc*, $HtrA^{S234A}$; black lines) were grown in C+Y medium at pHs 7.6 (top) and 7.3 (bottom). ADP309 shows a hypercompetent phenotype as described before (Stevens et al., 2011). Average of 3 replicates and Standard Error of the Mean (SEM) are plotted. **B) Growth curves (OD_{595}) and bioluminescence activity (RLU/OD_{595}) of the $HtrA^{S234A}$ strain in the presence of several antibiotics.** Strain ADP309 (P_{ssbB}-*luc*, $HtrA^{S234A}$) was grown in C+Y medium at pH 7.25, where it is not naturally competent anymore (black lines). Antibiotics used: 10 µg/ml gentamicin (GEN), 28 µg/ml tobramycin (TOB), 28 µg/ml aztreonam (ATM) and 2 µg/ml clavulanic acid (CLA). All antibiotics tested were able to induce competence, suggesting that ATM and CLA induce competence by another mechanism, independent of HtrA. Average of 3 replicates and Standard Error of the Mean (SEM) are plotted. Related to Figures 1 and 2.

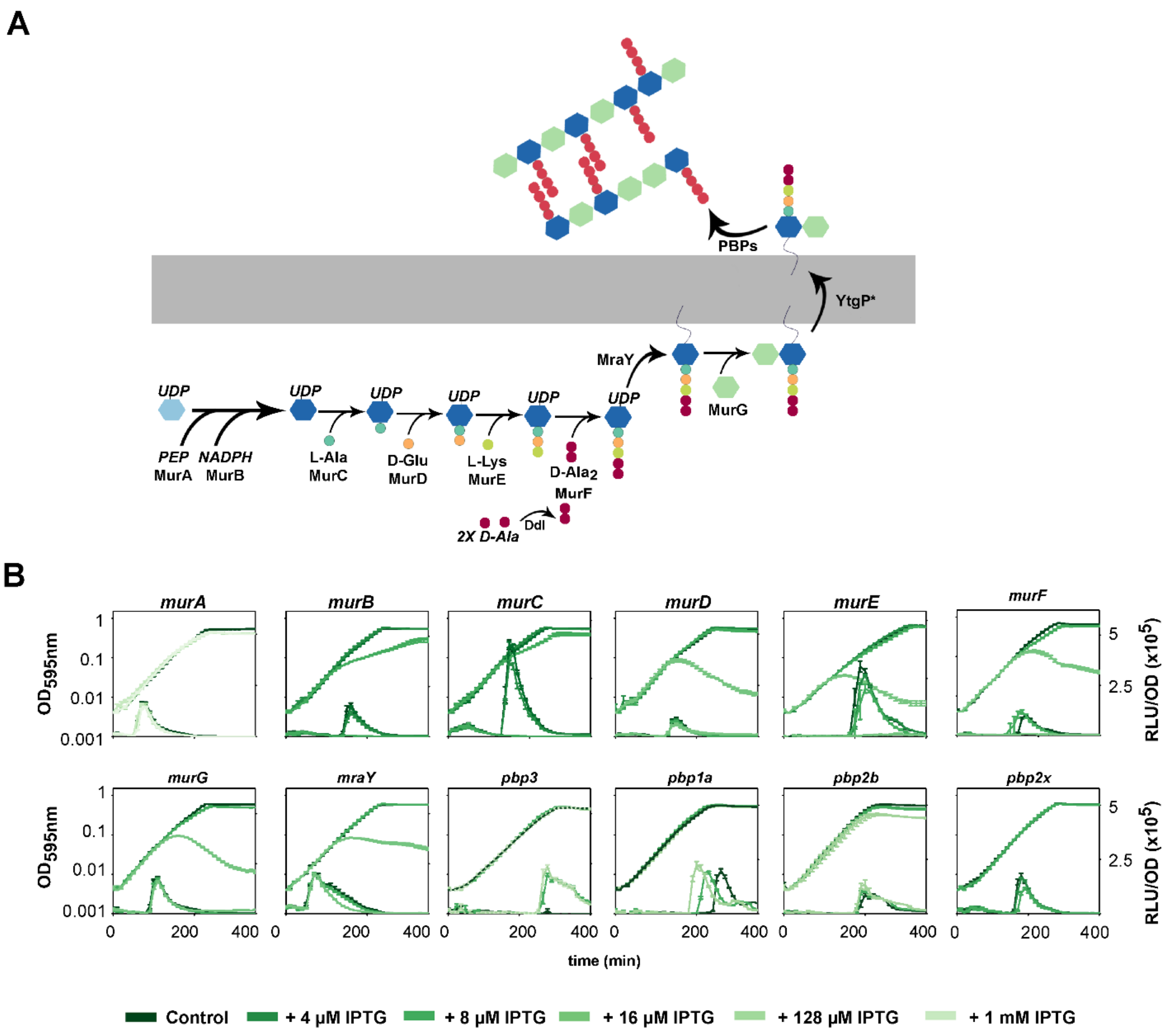

Figure S3. A) Representation of cell wall synthesis in *Streptococcus pneumoniae.* The first stage of peptidoglycan synthesis is the assembly of the pentapeptide precursor, executed by six enzymes (MurA-F). The second stage, which occurs on the intracellular part of the membrane, drives the synthesis of lipid II (MraY and MurG). Then, lipid II is flipped and exposed at the external part of the membrane, potentially by YtgP*.* The final stage occurs on the extracellular face of the membrane and involves the continuous transglycosylation and transpeptidation activities of the Penicillin-Binding Proteins (PBPs). **B) Repression of genes involved in pentapeptide (*murA-F*), lipid II (*murG* and *mraY*) formation and PBPs by CRISPRi.** No effect on competence was observed after depleting the individual genes with exception of *pbp1a and pbp3*. Detection of competence development was performed in C+Y medium at pH 7.5, permissive for natural competence. IPTG was added to the medium at the beginning, at different final concentrations (128 µM and 1 mM for *murA*; 4 µM and 8 µM for *murB* and *murC*; 8 µM and 16 µM for the other genes). Average of 3 replicates and Standard Error of the Mean (SEM) are plotted. Experiments were also reproduced at pH 7.3 (non-permissive) to confirm that the depletion of none of these genes causes an upregulation of competence (data not shown). Related to Figure 3.

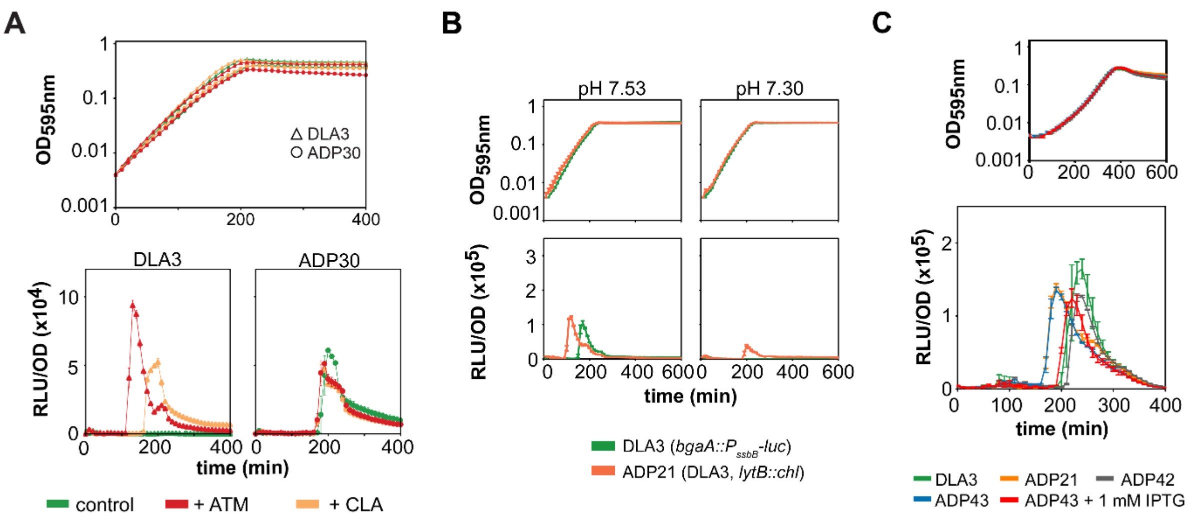

Figure S4. A) Natural competence in DLA3 (P$_{ssbB}$-*luc*) and ADP30 (P$_{ssbB}$-*luc, pbp3::chl*). To confirm that *pbp3* is involved in competence development, we compared the natural competence activation of the wild-type (DLA3, triangles) with the *pbp3* mutant (ADP30, dots). At pH 7.3, only the mutant was able to become competent (green), confirming the upregulation of this pathway in the absence of PBP3. In addition, competence is not further upregulated in the presence of aztreonam (ATM) or clavulanic acid (CLA) in the *pbp3* mutant. Average of 3 replicates and Standard Error of the Mean (SEM) are plotted. **B) Natural competence in DLA3 (P$_{ssbB}$-*luc*) and ADP21 (P$_{ssbB}$-*luc, lytB::chl*), in a range of three different pHs**. In all the conditions, the *lytB* mutant strain showed an earlier development of competence. Even at pH 7.3, where the wild-type strain did not become competent, the *lytB* mutant showed bioluminescence activity, indicating competence activation. Average of 3 replicates and Standard Error of the Mean (SEM) are plotted. **C) LytB complementation restores normal natural competence.** Strains showing a phenotype with many chains of cells [ADP21 (*lytB::chl*) and ADP43 (inducible *lytB* in *lytB::chl* background without presence of the inducer IPTG)] were hypercompetent compared with strains showing a wild-type phenotype [DLA3 (control), ADP42 (constitutive expression of LytB) or the induction of LytB in ADP43 with 1mM IPTG]. Cells were grown in C+Y at competence-permissive pH 7.6. Average of 3 replicates and Standard Error of the Mean (SEM) are plotted. Related to Figure 3.

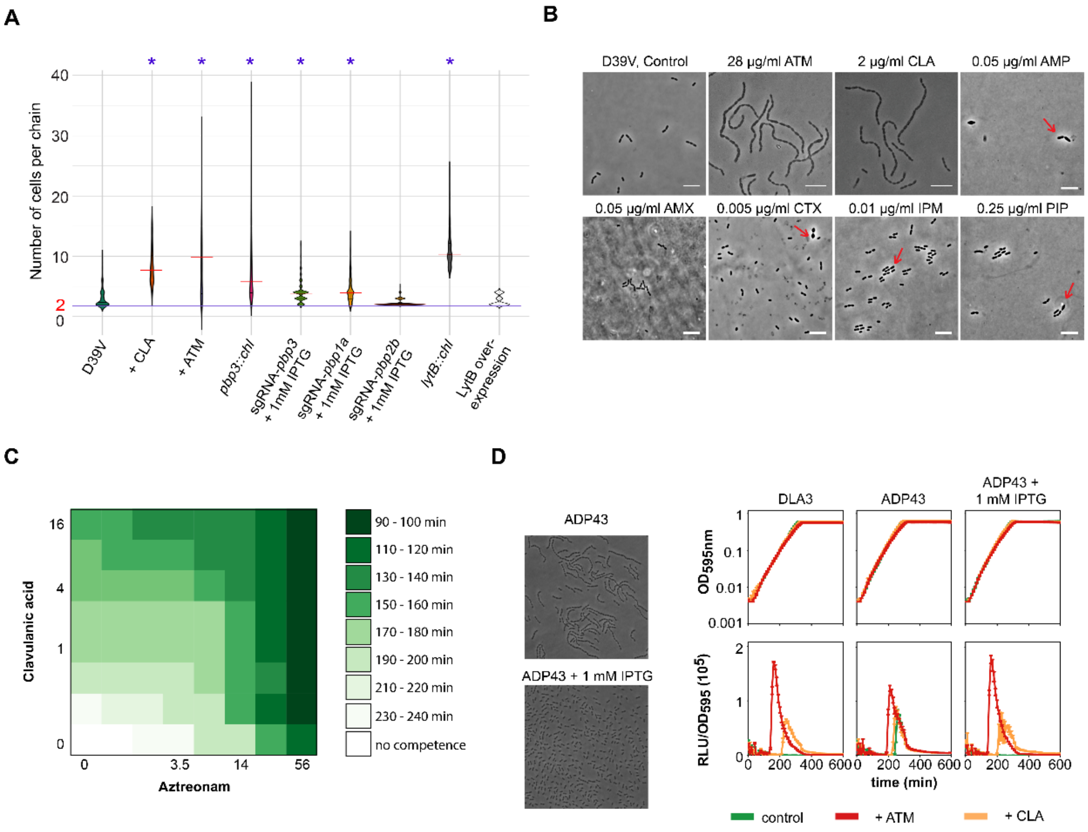

Figure S5. A) Length of the chains in cells growing in exponential phase. Horizontal red line indicates the average number of cells per chain while the purple line represents the typical diplococcus state. The addition of ATM or CLA results in the presence of longer chains, as does the deletion and depletion of *pbp3*. Depletion of *pbp1a* induces chain formation, contrary to the *pbp2b* depletion phenotype. The absence of *lytB* also resulted in an increase of chain length, while its overexpression restores normal chain length. Cell count for each condition: 2579, 2987, 4426, 7480, 1934, 1613, 1709, 5565 and 4012, respectively. *Statistically significantly longer chains than wild-type (mean comparison test, $p<0.05$). **B) Phase-contrast images of the D39V strain grown until OD$_{595nm}$ 0.1 in presence of several beta-lactams.** Cells were grown in C+Y acid medium (pH 6.8) until OD 0.1 (density at which cells become naturally competent in non-acidic conditions). Abbreviations: aztreonam (ATM), clavulanic acid (CLA), ampicillin (AMP), amoxicillin (AMX), cefotaxime (CTX), imipenem (IPM) and piperacillin (PIP). Only ATM and CLA induce chain formation. Scale: 6 µm. **C) Multi-dose-checkerboard of sub-inhibitory concentrations of aztreonam and clavulanic acid.** Values represent the first time point where the RLU value cells expressing luciferase from the *ssbB* promoter (strain DLA3) is ≥ 100 units in each condition. **D) Overexpression of LytB restores the diplococci phenotype and behaves as the wild-type strain.** Left, Microscopy of strain ADP43 (D39V, *bgaA*::P$_{ssbB}$-*luc*, *cep*::P$_{lac}$-*lytB*, *lytB::chl*, *prs1::lacI*) in the absence (top) or presence of 100 µM IPTG (bottom) at OD$_{595nm}$ 0.1. Right, effect of ATM and CLA in DLA3 and ADP43, in the absence or in presence of 1 mM IPTG at the non-permissive pH 7.3. In the absence of IPTG in strain ADP43, as chains are already there, the presence of ATM and CLA cannot induce more chains, so competence is only slightly accelerated (occurs approximately 10 - 20 minutes earlier than the strain without antibiotics). Furthermore, this strain is naturally hypercompetent, relative to the wild-type DLA3, which is not able to develop competence at this pH. However, the addition of IPTG in ADP43 restores the normal phenotype, and thereby, the strain behaves as DLA3: no competence activation in the control condition, and similar upregulation profiles by ATM and CLA due to the chaining-induced phenotype. Average of 3 replicates and Standard Error of the Mean (SEM) are plotted. Related to Figure 3.

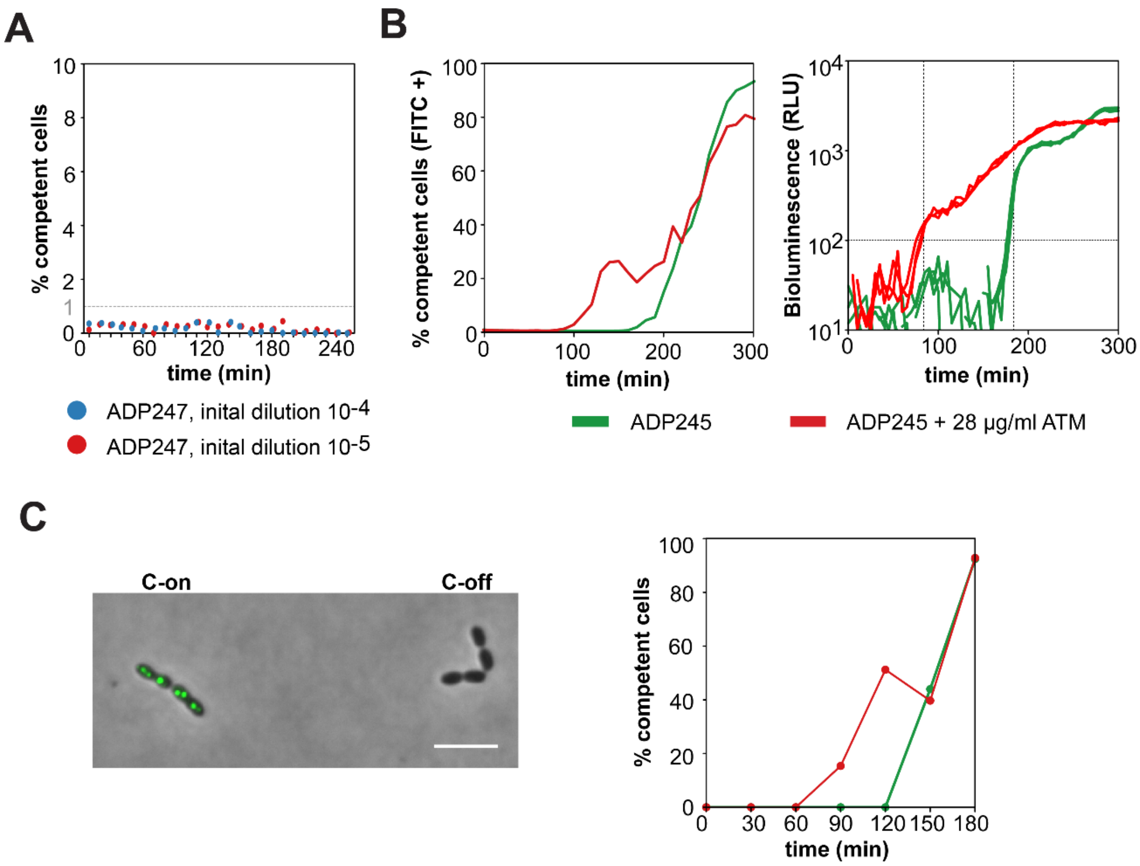

Figure S6. A) Estimation of the false positive rate in FACS experiments. A strain containing a translational fusion of GFP to the competence-induced SsbB protein (Aprianto et al., 2016), but lacking *comC*, was used as a negative control for our FACS experiments. This strain cannot become competent if there is no CSP present in the medium. Cells were grown at pH 7.8 and sampled (12000 particles per sample) every 10 minutes. The percentage of false-positive particles was below 1% of the population along the experiment, from two different initial dilution densities (10^{-4} in blue and 10^{-5} in red). **B)** Left, competence (FITC +) was induced earlier when aztreonam was present (red) than in the control condition (green). The slope of competence induction was drastically less steep with ATM, confirming a loss of synchronization within the population. Right, luminescence activity along the experiment. Vertical dashed lines show the first time point with GFP detection in presence (first line, 90 min) and absence (second line, 170 min) of ATM. Horizontal dashed line shows the cut-off of competence induction (100 RLU, established previously (Moreno-Gámez et al., 2017). The clear agreement between the flow cytometry (GFP) and luminescence data (*luc*) suggests that the luminometer used in all the experiments was able to accurately detect the timepoint where a significant portion of cells first became competent. **C)** Single cells were observed with fluorescence microscopy every 30 minutes, observing the same trend as in FACS experiments (panel A, center). An average of 250 cells were counted at every time point. White scale bar: 4 µm. C-on: competence upregulation. Note that in this experiment, cells were grown in C+Y at pH 7.9, to detect competence earlier than in Figure 4, to reduce the number of required reads in the FACS machine. Related to Figures 4 and 5.

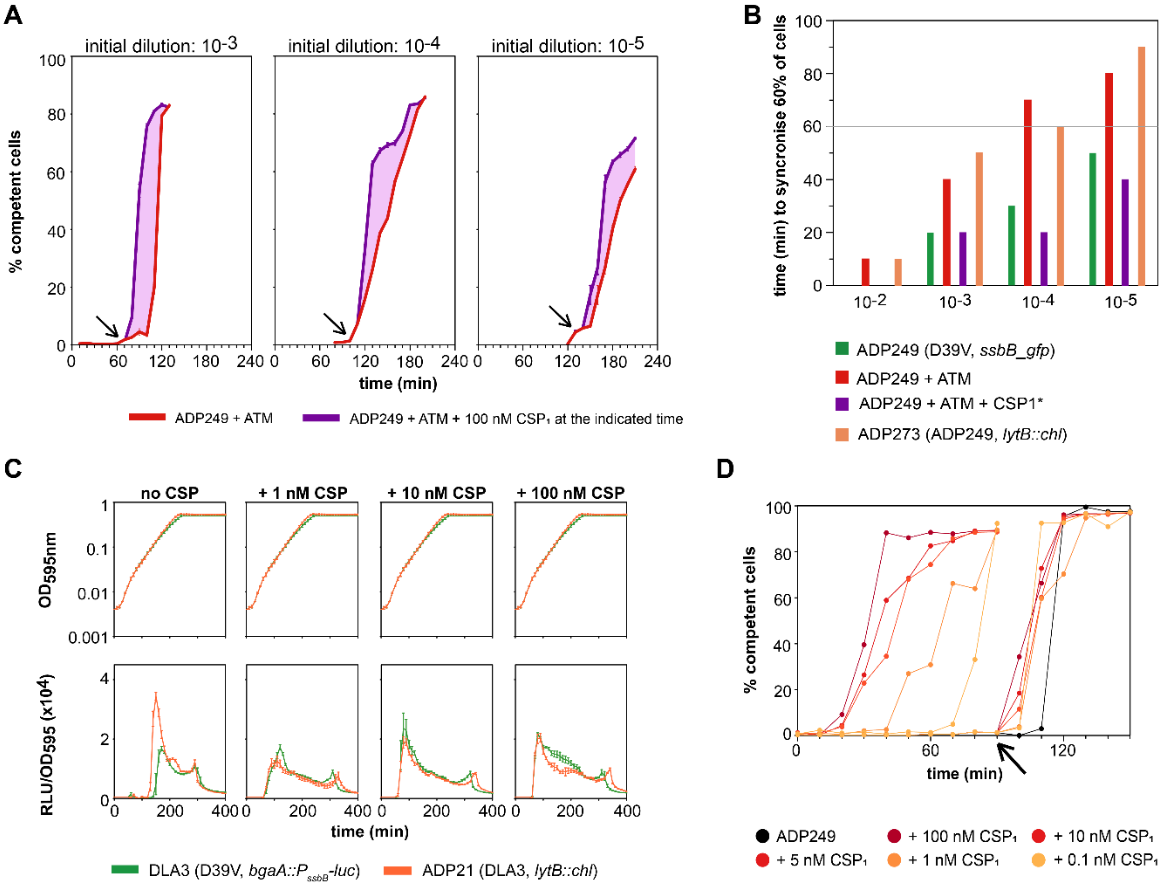

Figure S7. A) Competence synchronization in presence of aztreonam (ATM) and external CSP$_1$. Red lines show the percentage of competent cells over time in presence of 28 µg/ml of ATM; the data corresponds to Figure 5B). On the first positive value at each dilution (indicated by the arrows), 100 nM of synthetic CSP$_1$ was added to half of the remaining wells, and competence synchronization was tracked over time (purple line). The difference between presence or absence of CSP$_1$ is shown in pink. This data suggests that in the presence of ATM, competence signal propagation is slower by reduced CSP in the extracellular pool. Average of 3 replicates and Standard Error of the Mean (SEM) are plotted. **B) Lapse of time (minutes) between the first time point where competence was detected, and the value with $\geq$ 60% of competence in the overall population (data from Figures 5B and panel A).** In the initial dilution (10^{-2}), there is barely a difference between control (green), presence of ATM (red) or *lytB* mutant (orange), because there was no time to induce chain formation. Actually, green and purple bars for initial inoculum density 10^{-2} are missing as more than 60% of the cells were already competent in the first measurement. In contrast, in the other three initial dilutions, both ATM treatment and *lytB* deletion nearly doubled the time to synchronize 60% of the cells. The addition of exogenous 100 nM CSP$_1$ in the ATM condition (purple), reduced the synchronization time again to levels comparable to the control (data from panel A). **C) Addition of exogenous CSP$_1$ in wild-type and *lytB* mutant cells at the population level.** Cells were grown in C+Y at competence-permissive pH 7.6. After 60 minutes, the indicated concentration of CSP$_1$ was added. The average of 3 replicates and Standard Error of the Mean (SEM) are plotted for each condition. **D) Wild-type cells at the single-cell level.** ADP249 (*ssbB-gfp*) was grown in the presence or absence of exogenously added CSP$_1$. Cells were analyzed by flow cytometry every 10 minutes**.** A range of CSP$_1$ concentrations was added at two different time points: at the beginning and after 90 minutes (black arrow). Related to Figure 5.

Table S1. List of antibiotics tested for competence induction. Related to Figure 1.

Bacterial target	Antibiotic class	Antibiotic*	Concentration range tested (µg/mL)
Cell-wall inhibitors	Beta-lactams (carbapenems)	Imipenem	0.015 - 0.12
		Meropenem	0.015 - 0.12
	Beta-lactams (monobactams)	**Aztreonam**	12.5 – 100
	Beta-lactams (amino-penicillins)	Methicillin	0.016 - 2
		Ampicillin	0.0015 – 0.25
		Amoxicillin	0.0015 – 0.25
		**Amoxicillin/Clavulanic acid**	0.0015/2 – 0.25/2
	Beta-lactams (Cephalosporins)	Cephalexin (1st generation)	0.006 - 1
		Cefaclor (2nd generation)	0.006 – 1
		Cefuroxime (2nd generation)	0.006 – 1
		Cefotaxime (3rd generation)	0.006 – 1
		Cefepime (4th generation)	0.006 – 1
	Beta-lactams (antipseudomonal)	Piperacillin	0.003 – 0.12
	Beta-lactamase inhibitors	**Clavulanic acid**	0.5 – 8
DNA replication inhibition	Fluoroquinolones	**Ciprofloxacin**	0.4
	-	**HPUra**	0.15
	Aminoglycosides	**Gentamicin**	5 – 100

Protein synthesis inhibition			
		**Tobramycin**	5 – 100
	Macrolides	Clarithromycin (14 carbons)	0.003 – 0.12
		Azithromycin (15 carbons)	0.003 – 0.12
		Josamycin (16 carbons)	0.003 – 0.12
	Oxazolidinones	Linezolid	0.06 - 0.5

*In bold, antibiotics that induce competence in C+Y pH 7.3.

Table S2. Intraspecific *in vitro* horizontal gene transfer (HGT) of antimicrobial resistance determinants*. Related to Figure 2.

pH	Experiment	No. of transformants (cfu/ml)*	Total viable count (cfu/ml)	Transformation efficiency
**7.3**	DLA3 + MK134 (control)	0 ± 0	$1.4 \cdot 10^{11} \pm 4.0 \cdot 10^{10}$	0 ± 0
	DLA3 + MK134 + ATM	$6.2 \cdot 10^{4} \pm 4.1 \cdot 10^{3}$	$9.0 \cdot 10^{10} \pm 2.7 \cdot 10^{10}$	$7.0 \cdot 10^{-7} \pm 2.3 \cdot 10^{-7}$
	DLA3 + MK134 + CLA	$2.1 \cdot 10^{4} \pm 1.0 \cdot 10^{4}$	$9.3 \cdot 10^{10} \pm 1.5 \cdot 10^{10}$	$2.3 \cdot 10^{-7} \pm 1.4 \cdot 10^{-7}$
**7.5**	DLA3 + MK134 (control)	$2.0 \cdot 10^{3} \pm 1.0 \cdot 10^{3}$	$1.4 \cdot 10^{11} \pm 3.1 \cdot 10^{10}$	$1.3 \cdot 10^{-7} \pm 4.3 \cdot 10^{-9}$
	DLA3 + MK134 + ATM	$8.4 \cdot 10^{4} \pm 4.9 \cdot 10^{3}$	$9.0 \cdot 10^{10} \pm 2.6 \cdot 10^{10}$	$9.9 \cdot 10^{-7} \pm 3.0 \cdot 10^{-7}$
	DLA3 + MK134 + CLA	$5.3 \cdot 10^{4} \pm 7.5 \cdot 10^{3}$	$9.3 \cdot 10^{10} \pm 1.5 \cdot 10^{10}$	$5.9 \cdot 10^{-7} \pm 1.7 \cdot 10^{-7}$

* Three independent replicates per condition were performed. Strains: DLA3 (D39V, *bgaA::P$_{ssbB}$-luc*, tetracycline resistance marker), MK134 (D39V, *ssbB-luc*, kanamycin resistance marker). Abbreviations: ATM = 28 µg/ml of aztreonam, CLA = 2 µg/ml of clavulanic acid.

Table S3. Interspecific transfer of DNA from *E. coli* to *S. pneumoniae* promoted by aztreonam. Related to STAR methods.

Experiment	No. of transformants (cfu/ml)*	Total viable count (cfu/ml)	Transformation efficiency (%)
pLA18 *E. coli* + D39V SPNE	0 ± 0	$1.9 \cdot 10^{8} \pm 7.8 \cdot 10^{6}$	0 ± 0
pLA18 *E. coli* + D39V SPNE + ATM	$3.4 \cdot 10^{4} \pm 8.5 \cdot 10^{3}$	$1.0 \cdot 10^{8} \pm 3.5 \cdot 10^{6}$	$3.3 \cdot 10^{-4} \pm 9.3 \cdot 10^{-5}$

* Three independent replicates per condition were performed. Abbreviations: ATM: 28 µg/ml of aztreonam; SPNE: *Streptococcus pneumoniae*; *E. coli*: *Escherichia coli* DH5α carrying the high-copy plasmid pLA18, with the tetracycline resistance marker *tetM*. Both SPNE and *E. coli* were co-incubated at the same initial concentration (OD_{595nm} 0.04).

Table S4. Functional analysis of the microarray experiments of *S. pneumoniae* ADP62 (*comC::ery*) grown in presence or absence of antibiotics. Related to STAR methods.

Condition	Gene regulation	Class	Single list	Class Size	Description
ATM (RE)	upregulation	-			
	downregulation	COG	0.00 (3)	209	Cell wall/membrane/envelope biogenesis
		COG	0.00 (3)	159	Inorganic ion transport and metabolism
		GO	0.00022 (2)	7	GO:0005315 - phosphate transmembrane transporter activity
		GO	0.00051 (3)	92	GO:0006810 - transport
		GO	0.00194 (3)	164	GO:0016020 - membrane
		KEYWORDS	0.00258 (3)	121	IPR003439 - ABC transporter-like
		KEYWORDS	0.00082 (3)	66	IPR003445 - Cation transporter
ATM (AE)	upregulation	-			
	downregulation	COG	0.000 (3)	209	Cell wall/membrane/envelope biogenesis
		GO	0.00022 (2)	7	GO:0005315 - phosphate transmembrane transporter activity
		Others	0.0e+00 (4)	23	t_RNA-Ser
		Others	2.1e-06 (2)	2	Serine protease

CLA (RE)	upregulation	-			
	downregulation	-			
CLA (AE)	upregulation	-			
	downregulation	COG	0.00 (5)	209	Cell wall/membrane/envelope biogenesis
		COG	0.00 (4)	159	Inorganic ion transport and metabolism
		GO	7.4e-05 (2)	7	GO:0005315 - inorganic phosphate transmembrane transporter
		GO	0.0e+00 (4)	92	GO:0006810 - transport
		GO	0.0e+00 (5)	164	GO:0016020 - membrane
		IPR	0.00 (3)	37	IPR000515 - MetI-like domain
		KEYWORDS	0.0e+00 (4)	121	IPR003439 - ABC transporter-like
		KEYWORDS	6.9e-05 (3)	42	IPR000515 - MetI-like domain
		KEYWORDS	0.0e+00 (4)	66	IPR003445 - Cation transporter
		Pfam	0.00 (3)	37	PF00528 - Binding-transport system inner membrane
		Other	0.00 (3)	37	SSF161098 - MetI-like

Single list refers to the enrichment p-value, and between brackets, the number of genes in the toplists that are from that class. Class size means the total number of genes in that class. Abbreviations: ATM: aztreonam, CLA: clavulanic acid, RE: rapid exposure, AE: adaptive exposure; COG: clusters of orthologous groups, GO: gene ontology, IPR: interpro.

Table S5. Summary of gene expression changes in transcriptome comparison of *S. pneumoniae* D39V grown in presence *versus* absence of antibiotics. Related to STAR methods.

Condition	gene modulation	locus	gene description	Log2 fold change
ATM (RE)	downregulation	**SPD_0741**	Putative deoxyribose-specific ABC transporter permease protein	- 1.10
		**SPD_1231**	Phosphate transport system permease protein PstC2	- 1.12
		SPD_1393	pyridine nucleotide-disulfide oxidoreductase family protein	- 1.32
		**SPD_1230**	Phosphate transport system permease protein PstA2	- 1.35
		**SPD_0555**	Antibiotic ABC transporter permease protein	- 1.48
ATM (AE)	downregulation	SPD_1682	tRNA-Ser2	-1,01
		**SPD_1231**	Phosphate transport system permease protein PstC2	-1,01
		SPD_1695	tRNA-Leu3	-1,13
		SPD_1685	tRNA-Phe1	-1,13
		**SPD_1230**	Phosphate transport system permease protein PstA2	-1,19
		SPD_1760	tRNA-Cys1	-1,22
		SPD_2069	SpoJ protein	-1,34
		**SPD_0555**	Antibiotic ABC transporter permease protein	-1,36
		SPD_2068	Serine protease	-1,40

		SPD_0913	Hypothetical protein	-1,46
		**SPD_1697**	tRNA-Asp-GTC	-1,98
		SPD_1874	LysM domain-containing protein	-2,37
CLA (RE)	upregulation	SPD_0620	Lysyl-tRNA synthetase	2.31
		SPD_2028	Choline binding protein D	1.90
		SPD_1654	Ribosomal large subunit pseudouridine synthase B	1.08
		SPD_0919	Hypothetical protein	1.04
	downregulation	SPD_1697	tRNA-Asp-GTC	- 1.01
		SPD_0361	Transcriptional regulon	- 1.10
CLA (AE)	upregulation	SPD_1181	Hypothetical protein	1.19
		SPD_2007	Transporter major facilitator family protein	1.16
		SPD_0141	Hypothetical protein	1.04
		SPD_0293	PTS system transporter subunit IIA	1.02
		SPD_0934	Tn5252, ORF 10 protein	1.01
	downregulation	SPD_1738	MATE efflux family protein DinF	- 1.01
		SPD_1220	Spermidine/putrescine ABC transporter permease	- 1.01
		**SPD_0741**	Putative deoxyribose-specific ABC transporter permease protein	- 1.15
		**SPD_1231**	Phosphate transport system permease protein PstC2	- 1.23
		**SPD_1230**	Phosphate transport system permease protein PstA2	- 1.43

**SPD_0555**	Antibiotic ABC transporter permease protein	- 1.53
**SPD_1697**	tRNA-Asp-GTC	- 1.57

Abbreviations: ATM: aztreonam, CLA: clavulanic acid, RE: rapid exposure, AE: adaptive exposure. In bold, genes with transcriptome changes in more than one condition.

Table S6. List of strains used. Related to STAR methods*.

***S. pneumoniae* strains**	**Relevant genotype**	**Reference**
D39V	Serotype 2 strain	Avery et al. 1944
DLA3	Δ*bgaA::P$_{ssbB}$-luc*	Slager et al., 2014
MK134	*P$_{ssbB}$-ssbB-luc*	Slager et al., 2014
ADP21	Δ*bgaA::P$_{ssbB}$-luc, lytB::chl*	This study
ADP26	Δ*bgaA::P$_{ssbB}$-luc*, CPS*::chl*	Moreno-Gámez et al., 2017
ADP30	Δ*bgaA::P$_{ssbB}$-luc, dacC::chl*	This study
ADP42	Δ*bgaA::P$_{ssbB}$-luc, cep::P$_{Lac}$-lytB , lytB::chl*	This study
ADP43	Δ*bgaA::P$_{ssbB}$-luc, cep::P$_{Lac}$-lytB , lytB::chl, prs1::P$_{F6}$-lacI, P$_{ssbB}$-ssbB-luc*	This study
ADP62	Δ*bgaA::P$_{ssbB}$-luc, comC::ery*	Moreno-Gámez et al., 2017
ADP157	Δ*cep::P$_3$-sgRNA-pbp1A,* Δ*bgaA::P$_{Lac}$-dCas9, prs1::P$_{F6}$-lacI, P$_{ssbB}$-ssbB-luc*	This study
ADP161	Δ*cep::P$_3$-sgRNA-pbp2b,* Δ*bgaA::P$_{Lac}$-dCas9, prs1::P$_{F6}$-lacI, P$_{ssbB}$-ssbB-luc*	This study
ADP165	Δ*cep::P$_3$-sgRNA-murB,* Δ*bgaA::P$_{Lac}$-dCas9, prs1::P$_{F6}$-lacI, P$_{ssbB}$-ssbB-luc*	This study
ADP173	Δ*cep::P$_3$-sgRNA-murA-2,* Δ*bgaA::P$_{Lac}$-dCas9, prs1::P$_{F6}$-lacI, P$_{ssbB}$-ssbB-luc*	This study
ADP177	Δ*cep::P$_3$-sgRNA-pbp2x,* Δ*bgaA::P$_{Lac}$-dCas9, prs1::P$_{F6}$-lacI, P$_{ssbB}$-ssbB-luc*	This study

ADP178	*Δcep::P_3-sgRNA-mraY, ΔbgaA::P_{Lac}-dCas9, prs1::P_{F6}-lacI, P_{ssbB}-ssbB-luc*	This study
ADP179	*Δcep::P_3-sgRNA-murD, ΔbgaA::P_{Lac}-dCas9, prs1::P_{F6}-lacI, P_{ssbB}-ssbB-luc*	This study
ADP180	*Δcep::P_3-sgRNA-murG, ΔbgaA::P_{Lac}-dCas9, prs1::P_{F6}-lacI, P_{ssbB}-ssbB-luc*	This study
ADP187	*Δcep::P_3-sgRNA-murE, ΔbgaA::P_{Lac}-dCas9, prs1::P_{F6}-lacI, P_{ssbB}-ssbB-luc*	This study
ADP190	*Δcep::P_3-sgRNA-murF, ΔbgaA::P_{Lac}-dCas9, prs1::P_{F6}-lacI, P_{ssbB}-ssbB-luc*	This study
ADP203	*Δcep::P_3-sgRNA-murA-1, ΔbgaA::P_{Lac}-dCas9, prs1::P_{F6}-lacI, P_{ssbB}-ssbB-luc*	This study
ADP207	*Δcep::P_3-sgRNA-murC, ΔbgaA::P_{Lac}-dCas9, prs1::P_{F6}-lacI, P_{ssbB}-ssbB-luc*	This study
ADP247	*Δcep::P_3-mkate2, P_{ssbB}-ssbB-gfp, comC::ery*	Moreno-Gámez et al., 2017
ADP249	*Δcep::P_3-mkate2, P_{ssbB}-ssbB-gfp, bgaA::P_{ssbB}-luc*	Moreno-Gámez et al., 2017
ADP264	*Δcep::P_3-sgRNA-pbp3, ΔbgaA::P_{Lac}-dCas9, prs1::P_{F6}-lacI, P_{ssbB}-ssbB-luc*	This study
ADP273	*P_{ssbB}-ssbB-gfp, bgaA::P_{ssbB}-luc, lytB::chl*	This study
ADP305	*ΔbgaA::P_{ssbB}-luc,* PBP2X^{T550G}	This study
ADP306	*ΔbgaA::P_{ssbB}-luc, divIVA::ery*	This study
ADP308	*zip::P_{comC}-comC$^{'LP}$-hiBiT*	This study
ADP309	*bgaA::P_{ssbB}-luc, HtrA^{S234A}*	This study
ADP310	*ΔlytB::chl, zip::P_{comC}-comC$^{'LP}$-hiBiT*	This study
ADP311	*zip::P_{comC}-comC$^{'LP}$-hiBiT, comAB::ery*	This study

ADP312	*zip::P$_{comC}$-hiBiT*	This study
E. coli pLA18	*DH5α,* plasmid *amp^R, bgaA', tet^R, P$_{ssbB}$-luc_gfp, 'bgaA*	Slager et al., 2014

****ADP21 and ADP273 strains***: to monitor the effect of chain formation on competence induction, the gene *lytB*, encoding Autolysin B, was replaced by the chloramphenicol resistance marker. The upstream region was amplified using primers ADP1/34 (GATGTGGTGAAAGCAGCTGTGGAAG) and ADP1/35+AscI (CGATGGCGCGCCTCCTCTGTTCTTATTTATTTTATTG), the downstream region with primers ADP1/36+NotI (CGATGCGGCCGCTACTATAAGTGAATATGATTTGAGTG) and ADP1/37 (GTGTAGAAACCGTCCTCAACCAAG), and the chloramphenicol resistance marker with sPG11+AscI (ACGTGGCGCGCCAGGAGGCATATCAAATGAAC) and sPG12+NotI (ACGTGCGGCCGCTTATAAAAGCCAGTCATTAG). All three fragments were digested with the proper restriction enzymes (AscI and/or NotI) and ligated together. The Δ*lytB::chl* fragment containing the chloramphenicol resistance marker flanked by the sequence up- and downstream of *lytB* was transformed into DLA3 resulting in ADP21 strain (Δ*bgaA::P$_{ssbB}$–luc,* Δ*lytB::chl*), and into ADP249 resulting in ADP273 strain (Δ*bgaA::P$_{ssbB}$–luc, P$_{ssbB}$–ssbB-gfp,* Δ*lytB::chl).* Transformants were selected on Columbia blood agar containing 4.5 µg/ml chloramphenicol. Correct deletion was verified by PCR and sequencing. ***ADP30 strain***: to monitor the influence of PBP3 on competence, the related gene was replaced by the chloramphenicol resistance marker. The upstream region was amplified using primers ADP1/59 (GCCCTCAACTCAGCAGTATGG) and ADP1/60+AscI (CGATGGCGCGCCTTATCCAACTGATCCCTCCATTTC), the downstream region with primers ADP1/61+NotI (CGATGCGGCCGCGAGGTAACTCATGTTTCGTAG) and ADP1/62 (AAGCCTGCAATATGCAAGCGATCC), and the chloramphenicol resistance marker with sPG11+AscI (ACGTGGCGCGCCAGGAGGCATATCAAATGAAC) and sPG12+NotI (ACGTGCGGCCGCTTATAAAAGCCAGTCATTAG). All three fragments were digested with the proper restriction enzymes (AscI and/or NotI) and ligated. The Δ*pbp3::chl* fragment containing the chloramphenicol resistance marker flanked by the sequence up- and downstream of *pbp3* was transformed into DLA3 resulting in ADP30 strain (Δ*bgaA::P$_{ssbB}$–luc,* Δ*pbp3::chl*). Transformants were selected on Columbia blood agar containing 4.5 µg/ml chloramphenicol. Correct deletion was verified by PCR and sequencing. ***ADP42 and ADP43 strains***: to test whether the ectopic hyperexpression of LytB in the Δ*lytB* mutant restored the normal diplococcus phenotype and restored competence development to wild type, we created an inducible expression of LytB. The inducible system was created using BglFusion cloning (Sorg et al., 2015). To amplify the *lytB* fragment, primers ADP1/71+BglII (ACGTAGATCTAGAGGAAGAAGGTTGATGAAGAAAG) and ADP1/72+XhoI (CATGCTCGAGTTACTGGAGGGATCCAGTACTAATCTTTG) were used with D39V chromosomal DNA as a template. The construction was transformed into strain ADP21 and transformants (ADP42) were selected on Columbia blood agar containing 100 µg/ml spectinomycin. Correct deletion was verified by PCR and sequencing. ADP42 shows constitutive expression of *lytB* since it lacks the LacI repressor of the IPTG-inducible system. To control the expression of LytB, we then transformed the codon-optimized *lacI* gene into strain ADP42. For that, we PCR-ed the fragment with *lacI* integrated into the *prs1*/*prsA*-locus together with a gentamycin resistance cassette from chromosomal DNA of strain ADP95 (Moreno-Gámez et al. 2017), using primers OLI40 (CCATGGCATCAGCGAGAAGGTGATAC) and OLI41 (GCGGCCGCAGGATAGAAAGGCGAGAG). ***CRISPRi library:*** to monitor the effect on competence of the downregulation of genes involved in the cell wall synthesis, the PCR product of the fragment *P$_{ssbB}$-ssbB–luc–kan* from strain MK134 (Slager et al. 2014) was transferred into the CRISPRi library of the indicated genes (Slager et al. 2014; Liu et al. 2017). Transformants were selected on Columbia blood agar containing 250 µg/ml kanamycin. ***ADP305 strain:*** to test whether ATM and CLA induce competence in a strain with reduced susceptibility to beta-lactams, we have introduced a point mutation in the pbp2X (PBP2X^{T550A}), which increases the MIC to cefotaxime from 0.02 µg/ml to 0.65 µg/ml. To do so, we have overlap the PCR products obtained with primers: RS6/31 (CCGAATTGGACGATGCCAAG) and OVL1220 (CGTCAGCAATCTGAGCTCCACCAGACTTGAGGGCTAC), and with ADP4/5 (CAGTGCATGCCTTACATCAAATACAAAATTGCGAGG) and OVL1219 (GTAGCCCTCAAGTCTGGTGGAGCTCAGATTGCTGACG). Overlapped fragment was transformed into DLA3 (Δ*bgaA::P$_{ssbB}$–luc).* Transformants were selected on Columbia blood agar containing 0.1µg/ml cefotaxime. Correct construct was verified by PCR and sequencing. ***ADP308, ADP310 and ADP311 strains.*** HiBit construct was designed by fusing the C-terminus of the region of interest with the 11-amino acid HiBiT peptide using a 10-amino acid linker. The region of interest was the putative secretion signal (until the double glycine) of *comC*. The expression of these constructs was designed to be controlled by the *comC* promoter region. The construct (Gblock, IDTlab) was cloned in the pPEPZ plasmid and introduced in D39V strain resulting in ADP308 strain. The PEPZ plasmid integrates at the non-coding gene SPV_1735 under *zip* name (pPEP**Z** **I**ntegration **P**osition). Transformants were selected on Columbia blood agar containing 100 µg/ml spectinomycin. PCR products of *lytB::chl* (from ADP21, described above) or *comAB::ery* were introduced to ADP308 resulting in strains ADP310 and ADP311, respectively. ***ADP312 strain.*** To confirm that HiBiT luminescence is due to the export of the peptide by ComAB rather than the release by cell lysis, we designed the

HiBiT sequence to be controlled by the *comC* promoter, without the leader peptide. Hence, once HiBiT is produced, is accumulated in the cytoplasm. To do so, we overlap the ADP308 construct (P_{comC}-*comC'*^{LP}-*hiBiT*) with primers OVL2164 (GAAAAAACATTTTAGGAGATTTTATTATGGGTGGTGGTGGTTCTGGTGG) and OVL2165 (CCACCAGAACCACCACCACCCATAATAAAATCTCCTAAAATGTTTTTTC) to remove the leader peptide sequence. Overlapped fragment was transformed into D39V strain.
